# Systematic analysis of the interaction mechanism between platelets and coronary heart disease: from molecular pathways to new strategies for plant based antiplatelet therapy

**DOI:** 10.3389/fphar.2025.1586265

**Published:** 2025-06-03

**Authors:** Li Liao, Xiaoxuan Li, Hong Xu, Jing Tang, Bo Li, Yan Tang, Fang Xie

**Affiliations:** ^1^ Pharmaceutical Department, The Second People’s Hospital of Yibin-Yibin Hospital of West China Hospital of Sichuan University, Yibin, China; ^2^ Clinical Pharmacy Department, Third Affiliated Hospital of Chengdu Medical College-Chengdu Pidu District People’s Hospital, Chengdu, China; ^3^ Department of Oncology, The Second People’s Hospital of Yibin-Yibin Hospital of West China Hospital of Sichuan University, Yibin, China

**Keywords:** coronary heart disease, platelets, thrombus, inflammation, traditional Chinese medicine

## Abstract

Coronary Heart Disease (CHD) stands as a predominant cardiovascular ailment globally, posing a severe menace to human health. Central to both hemostasis and the pathogenesis of coronary atherosclerotic thrombosis are platelets. In recent years, their significance has expanded beyond mere involvement in clot formation; they have been implicated in heightened immune responses, contributing to tissue inflammation (evident in myocardial ischemia) and vascular inflammation (crucial in vulnerable plaque formation). While contemporary antiplatelet therapies have markedly enhanced clinical outcomes for patients with coronary artery disease, they inadvertently escalate the hazard of bleeding complications. This review delves into the intricate mechanisms by which platelets influence the progression of coronary artery disease and meticulously examines the prospective utility of herbal antiplatelet interventions. Our objective is twofold: firstly, to furnish clinicians with scientifically grounded and valuable therapeutic alternatives for managing coronary artery disease, and secondly, to stimulate research and development endeavors aimed at creating novel, more efficacious antiplatelet medications that strike a balance between efficacy and safety.

## 1 Introduction

Coronary heart disease (CHD), also known as coronary atherosclerotic heart disease, is a cardiovascular disorder characterized by the gradual narrowing of coronary arteries due to plaque accumulation or atherosclerosis (Baaten et al., 2024). Its hallmark feature is the substantial obstruction within the vascular lumen, which not only results in myocardial ischaemia and hypoxiabut can also precipitate cardiomyocyte necrosis. These pathological alterations ultimately culminate in severe outcomes such as heart failure, arrhythmias, and even sudden cardiac death (Dong et al., 2019). Currently, CHD ranks among the leading causes of mortality and morbidity worldwide (Bi et al., 2015).

Platelets, multifunctional nucleate cells with a diameter of 2–4 μm, are present at concentrations of 150–350 × 10^9^/L in healthy individuals. Despite lacking genomic DNA, they contain megakaryocyte-derived messenger RNAs (mRNAs) and translational machinery essentialfor protein synthesis (Davizon-Castillo et al., 2020). Platelets play a critical role in maintaining vascular wall integrity, preventing excessive blood loss due to tissue damage (de Gaetano and Cerletti, 2002; Brewer, 2006), and are central to processes such as thrombosis and haemostasis (Davì and Patrono, 2007). Consequently, platelets are increasingly recognised as key players in many other pathophysiological processes, including inflammation, atherosclerosis, obstructive and non-obstructive coronary artery disease (CAD), and a wide range of serious diseases such as ischemic stroke (Sabetta et al., 2022). Antiplatelet therapy is therefore considered one of the most important tools for the prevention of cardiovascular diseases such as ischemic heart disease (IHD) (Wiviott et al., 2007; Wallentin et al., 2009), stroke (Amarenco et al., 2020), and peripheral arterial disease (PAD) (Bonaca et al., 2013).

In traditional medicine, CHD is classified under the conditions of chest paralysis and heart pain. Its etiology primarily encompasses factors such as qi depression, phlegm, blood stasis, fire-heat, cold condensation and zhengqi deficiency. The pathological mechanisms mainly involve processes like coagulation, inflammation and immune response (Lan et al., 2024). As a traditional medical resource mainly derived from natural herbs, Traditional Chinese Medicine (TCM) boasts advantages such as multi-targeting, multi-pathway, low adverse effects and a wealth of clinical practice, has long been employed to maintain human health. At present, a multitude of published randomised controlled trials (RCTs), systematic evaluations and meta-analyses have affirmed that TCM offers certain benefits in the comprehensive prevention and control of coronary artery lesions (Teng et al., 2024). It can not only effectively impede early critical lesions but also delay the onset of heart failure complications following advanced myocardial infarction (Cheang et al., 2024; Chen K. et al., 2023). Furthermore, TCM improves symptoms associated with CHD like chest tightness and chest pain (Zhang et al., 2025). Hence, it is crucial to investigate the pharmacological and material basis of anti-CHD treatments and their related mechanism of action within the framework of TCM.

## 2 Mechanism of platelets in the development of CHD

CHD is characterized by myocardial ischemia resulting from coronary artery atherosclerosis. It frequently manifests with symptoms such as angina pectoris and can lead to severe complications, including myocardial infarction. Given the critical nature of this condition, it becomes imperative to understand the role of platelets in its pathogenesis. What, then, is the contribution of platelets to the development and progression of CHD?

### 2.1 Platelets and thrombosis

As is well known, during normal blood circulation, the vascular wall remains intact and platelets remain in a non-activated state, maintaining the integrity of the vascular endothelium (Knowles and Warner, 2019). However, when platelets are activated, they will further adhere and aggregate, forming thromboembolic bodies, leading to the occurrence of diseases related to arterial thromboembolism such as ischemic stroke and coronary heart disease (Chen K. et al., 2023). For example, clinical study in the context of acute coronary syndrome (ACS), there is a notable alteration in the platelet lipidome, which not only stimulates platelet activation but also accelerates the progression of ACS by promoting thrombotic events (Harm et al., 2022).

When the vascular wall is damaged by external factors or pathological stimuli, the activated thrombin or exposed subendothelial matrix produced after endothelial injury can trigger platelet activation and aggregation, leading to changes in platelet biochemistry and structure and stimulating platelet activation. Subsequently, activated platelets adhere and aggregate through a series of reactions, forming an embolic body. For example, research has demonstrated that the platelet transmembrane chemokine SR-PSOX/CXCL16-CXCR6 synergistically accelerates the release of platelet degranulation and platelet aggregation to promote thrombosis in CAD (Guan et al., 2022). In turn, dense granules and α-granules released by activated platelets further amplify platelet activation and promote thrombosis via the release of adenosine triphosphate (ATP) or adenosine diphosphate (ADP) and complement factors (Stenberg et al., 1984). Concurrently, activated platelets also lead to the exposure of negatively charged phospholipids on the surface, which stimulates the binding and activation of coagulation and tissue factors, further promoting thrombosis (Gremmel et al., 2024); and its derivative proprotein convertase subtilisin/kexin type 9 (PCSK9) also induces a platelet-dependent thrombotic inflammatory response and promotes atherosclerotic thrombosis (Petersen-Uribe et al., 2021). Furthermore, inhibition of cystic fibrosis transmembrane conductance regulator protein (CFTR) in platelets also enhances platelet activity and accelerates arterial thrombosis by increasing intracellular Cl^−^ concentration and promoting the recombinant serum/glucocorticoid regulated kinase 1 (SGK1) signaling pathway (Yang et al., 2022). In general, activated platelets promote thrombus formation through the following mechanisms: (1) activation of integrin αⅡbβ3 (signal transduction from the inside out), binding to fibrinogen, fibronectin, or VWF, and aggregating and adhering at the damaged site (Grover et al., 2018); (2) Promote thrombin activation, initiate coagulation cascade reaction, and form stable and irreversible thrombus (Furie and Furie, 2008); (3) By increasing intracellular Ca^2+^concentration, bioactive molecules such as adenosine diphosphate (ADP), P-selectin, thromboxane A2 (TXA2), and serotonin (5-HT) are released (Jurk and Kehrel, 2005), further enhancing platelet aggregation and forming firm embolic bodies (Kubes, 2016), thereby promoting the progression of cardiovascular disease.

Therefore, platelet activation plays a crucial role in occurrence, development and prognosis of CHD by facilitating thrombosis.

### 2.2 Platelets and vascular endothelial function

The vascular endothelium comprises extremely thin vascular endothelial cells that serve as a mechanical barrier between the vessel wall and the bloodstream. These cells selectively regulate the passage of substances of various sizes through the vessel wall, as well as modulate vascular smooth muscle contraction, platelet aggregation, vascular smooth muscle cell proliferation, leukocyte adhesion, and thrombus formation. Consequently, damage to the vascular endothelium and subsequent inflammatory responses are significant contributors to the development of CHD (Roth et al., 2020; GBD 2019 Diseases and Injuries Collaborators, 2020). In general, endothelial cells (ECs) covering the vascular lumen protect vascular integrity and homeostasis by sensing and responding to physical, chemical, and biological stimuli (Feaver et al., 2013; Giannotta et al., 2013). When exposed to external stimuli such as high/low shear forces, these cells undergo major phenotypic changes, leading to increased endothelial cell permeability, cytokine release, and leukocyte adhesion (Ni et al., 2010; Gimbrone, 2010), ultimately resulting in endothelial cell damage. In addition, the imbalance between the generation and accumulation of ROS/free radicals and the ability of antioxidants (such as superoxide dismutase, glutathione peroxidase, catalase, vitamin E) to clear them can lead to oxidative stress, resulting in cell damage (Pizzino et al., 2017), thus leading to the onset and progression of atherosclerotic cardiovascular disease.

Studies have shown that platelets rapidly recognize and respond to damage in the vascular system during injury, further activating it and affecting endothelial cell function by releasing a variety of cytokines. For instance, platelets stimulate the synthesis and release of interleukin-1α (IL-1α) (Thornton et al., 2010), interleukin-1β (IL-1β) (Lindemann et al., 2001) and chemokines such as monocyte chemotactic protein 1 (MCP-1) (Blair and Flaumenhaft, 2009; Pennings et al., 2022). They also promote interactions between platelets and leukocytes, neutrophils, and endothelial cells (Gawaz et al., 2005; Merhi et al., 1995), which exacerbating plaque rupture and thrombotic inflammatory responses (Bakogiannis et al., 2019; Steg et al., 2011). Concurrently, activated platelets can also directly adhere to endothelial cells following thrombin stimulation (Kaplan et al., 1989); or bind to Willebrand factor (vWF) via glycoprotein (GP)Iβα on their surface before adhering to endothelial cells (Auid-Orcid Zifkos et al., 2024), ultimately intensifying inflammatory injury. The stable binding of platelets to vWF further stimulates collagen interacts with GPVI and α2β1 integrin. This interaction triggers platelet activation, leading to inside-out signaling, which then activates Glycoprotein IIb/IIIa (GPIIb/IIIa), allowing it to bind fibrinogen and von vWF for platelet aggregation (Denis and Wagner, 2007). This, in turn, promotes the cytosolic action of dense granules such as ADP, ATP, Ca^2+^ and 5-hydroxytryptamine (5-HT), thereby enhancing platelet aggregation and activation at sites of vascular injury (Fraer and Kilic, 2015). Additionally, platelet-expressed toll-like receptor 2 (TLR2) boost platelet activity and interactions with vascular endothelial cells, contributing to the development of thrombo-inflammatory disease (Parra-Izquierdo et al., 2021).

These findings suggest that platelet activation and vascular endothelial cell adhesion can induce a vascular inflammatory response, which in turn accelerates the progression of cardiovascular disease (Badimon et al., 2012).

### 2.3 Platelets and inflammatory response

The immune system plays a central role in atherosclerotic thrombosis (Verdoia et al., 2015). In contrast, the inflammatory response is a catalyst for CHD disease progression.

In acute myocardial infarction, activated platelets release transforming growth factor B-inducible (TGFBI) protein, which stimulates platelet activation, adhesion, migration, and vascular inflammation, thereby contributing to the disease progression of myocardial infarction (MI) (Kraemer et al., 2022). At the same time, these activated platelets also release 5-HT or serotonin (Mauler et al., 2019), inducing neutrophil degranulation and the release of myeloperoxidase, hydrogen peroxide (H_2_O_2_), and the membrane-bound leukocyte adhesion molecule recombinant integrin alpha M (CD11b). This ultimately leads to enhanced inflammation in the infarcted area and reduced myocardial salvage. Furthermore, platelet-neutrophil aggregates formed by activated platelets and neutrophils can contribute to the development of various thrombotic-inflammatory disorders (Zarbock and Ley, 2009; Schmitt et al., 2015). Additionally, platelet microvesicles (PMV), ultramicrofilm vesicle released by platelets during activation, can also exacerbate the vicious cycle of inflammation and thrombosis during ACS (Gkaliagkousi et al., 2021). In ApoE^−/−^mice, platelet-released P-selectin facilitates the delivery of platelet-derived pro-inflammatory factors to monocytes/leukocytes and the vessel wall, thereby inducing an inflammatory response and plaque enlargement (Huo et al., 2003). Additionally, platelet surface CD154 can also accelerate atherosclerosis by inhibiting regulatory T cells and reducing plaque stability (Lievens et al., 2010).

However, it has also been shown that soluble guanylate cyclase (sGC) in platelets can inhibit atherosclerotic plaque formation by stimulating the release of angiopoietin-1 from platelets and reducing leukocyte recruitment *in vitro* (Mauersberger et al., 2022). Platelet-derived miRNAs can ameliorate myocardial inflammation following myocardial ischemia-reperfusion by inhibiting myocardial inflammation and cardiac fibrosis (Schütte et al., 2023). Additionally, platelet microparticles containing miR-4306 can also improve CAD by inhibiting human monocyte-derived macrophage migration via the VEGFA/ERK1/2/NF-κB signaling pathway (Yang et al., 2019). In addition, miR-34c-5p derived from extracellular vesicles of activated platelets (PLT-EVs) also attenuates inflammatory responses in HCAEC through inhibition of the podocalyxin (PODXL) and P38 MAPK signaling pathways, thereby preventing the development of CAD (Bai et al., 2022).

**FIGURE 1:**
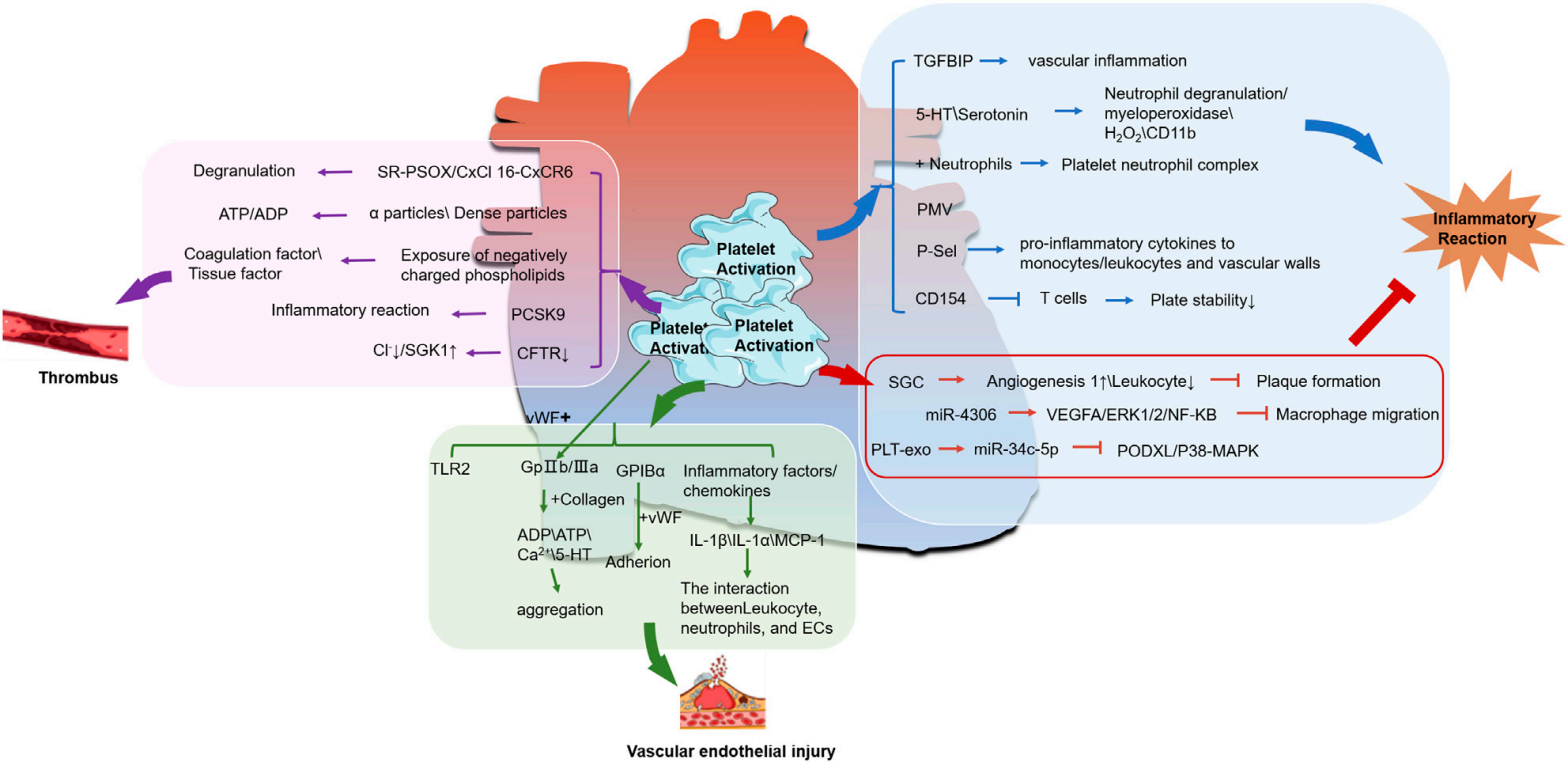
The relationship between activated platelets and CHD. (The molecular mechanism between platelet activation and thrombus formation is shown in the pink box; The green box represents the relationship between platelet activation and endothelial injury, while the blue box represents the molecular mechanism between platelet activation and inflammatory response. ↑ is upregulation, ↓ is downregulation, 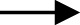 is promotion, 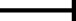 and is inhibit).

Collectively, these studies indicate that platelets possess the dual capacity to both enhance and suppress inflammatory responses via a multitude of pathways, as illustrated in Figure 1. Consequently, they play a significant role in influencing the progression and outcome of atherosclerotic disease.

## 3 Progress in the study of antiplatelet effects in TCM

At present, representative drugs widely used in clinical practice for the treatment and prevention of thrombotic diseases, such as commonly used antiplatelet drugs (aspirin, clopidogrel, and GPIIbIIIa inhibitors) (Mega and Simon, 2015), often come with side effects such as drug resistance (Rozalski et al., 2005), intolerance, allergies, “aspirin resistance”, and gastrointestinal bleeding (Angiolillo, 2012). TCM is considered by scholars as a new alternative therapy (Li et al., 2019). TCM, primarily derived from natural herbs, has a long history in treating thrombotic diseases. Modern pharmacological studies have demonstrated that TCM are effective in antithrombotic therapy, particularly in inhibiting platelet activity (Qiu et al., 2019; Zhou et al., 2019), such as plant extracts ketones, alkaloids, and alcohol glycosides, all have antiplatelet activity, promote fibrinolysis, and anticoagulant activity (Wei et al., 2020).

### 3.1 Inhibition of platelet adhesion

Platelet adhesion refers to the adhesion of platelets to the surface of subendothelial tissues or other substances in blood vessels. It is the initial reaction of normal hemostasis after vascular damage. Studies have shown that the release of P-selectin from stimulated endothelial cells mediates the initial loose contact between platelets and endothelial cells (Frenette et al., 1998; Frenette et al., 1995), which in turn affects the progression of CHD.

In the study of herbal extracts, Saccharides from *Arctium lappa* L. root, inhibits platelet spreading on fibrinogen and adhesion on collagen under shear (Ruan et al., 2022).

Regarding the single components of TCM, research has demonstrated that the combination of total saponins from the stem and leaves of *Panax quinquefolius* L. and total saponins from PNF attenuates injured endothelium-induced platelet adhesion by up-regulating the PI3K/PKB pathway in the endothelial cells (Ming-ming et al., 2016). Panax notoginseng saponin inhibits low shear stress-induced platelet adhesion and activation on dysfunctional endothelial cells (Liu et al., 2024). Panax notoginseng triol saponin reduces vascular VWF-mediated platelet adhesion to damaged vascular endothelium, enhancing the potential for antiplatelet aggregation and antithrombosis under pathological conditions (Xu et al., 2021). Salvianolic acid B from *Salvia miltiorrhiza* Bge, inhibits platelet adhesion to immobilized collagen by interfering with the collagen receptor α2β1 (Wu et al., 2008). It also reduced platelet adhesion to endothelial cells and platelet aggregation to leukocytes (Li et al., 2025). Epigallocatechin-3-gallate, found in high levels in dried green tea leaves, has been shown to inhibit platelet adhesion and aggregation (Joo et al., 2018).

In addition, the Chinese medicine compound preparation QiLong capsule reduces platelet adhesion by interfering with platelet CD36 expression and affecting the level of β-thromboglobulin (β-TG), PF-4, P-selectin and platelet activating factor (PAF) (Wang et al., 2024). Gegen Qinlian pills also inhibit platelet aggregation and adhesion by regulating HMGB1/NF-κB/NLRP3 signaling pathway and attenuating keratine-induced thrombosis in mice (Wei et al., 2022).

In summary, TCM exhibit significant inhibitory effects on platelet activity. These effects can be effective in both the prevention and treatment of cardiovascular diseases through mechanisms such as anti-platelet aggregation, adhesion, and inhibition of platelet content release, as illustrated in Table 1.

**TABLE 1:** Researches on TCM inhibit platelet adhesion.

Category	Source	Active chemical	Model	Doses	Effects	Pathway	Reference
Extracts	*Arctium lappa* L.	Saccharides	*In vitro* platelet activation model (induced by Collagen, thrombin, ADP) and mouse carotid artery thrombosis model induced by FeCl_3_	20, 60, 200 (µg/mL)	Inhibit platelet spreading on fibrinogen and adhesion on collagen, and attenuated platelet activation	Oxidative stress	Ruan et al. (2022)
Monomer compound	*Salvia miltiorrhiza* Bge.	Salvianolic acid B	—	500, 1,000 (μg/mL)	Inhibit platelet adhesion by interfering with the collagen receptor α2β1	Collagen receptor α2β1	Wu et al. (2008)
	*Salvia miltiorrhiza* Bge.	Salvianolic acid B	*In vitro* platelet activation model	20 (mg/kg)	Reduce platelet adhesion to endothelial cells and platelet aggregation to leukocytes	Platelet receptor CD226	Li et al. (2025)
	*Camellia sinensis* (L.) Kuntze	Epigallocatechin-3-gallate	*In vitro* platelet activation model (induced by Collagen, thrombin receptor activating peptide (TRAP), ADP)	50, 100, 150, 200 (μM)	Inhibited platelet adhesion and aggregation	Shear stress	Joo et al. (2018)
	*Panax notoginseng* (Burkill) F. H. Chen	Panax notoginseng triol saponins	Middle cerebral artery occlusion model	25, 50, 100 (mg/kg)	Inhibit platelet aggregation through regulating GP1BA	GP1BA	Xu et al. (2021)
PolyPill	Gegen Qinlian pills	—	*In vitro* platelet activation model (induced by thrombin, ADP)	1, 2, 5 (μg/mL)	Reduce inflammation-induced thrombosis	HMGB1/NF-κB/NLRP3	Wei et al. (2022)
	Qilong capsule	—	platelet activation model (induced by ADP, AA) and Cardiac ischemia‒reperfusion injury model (Wang et al., 2020)	0–25 (mM), 0.2, 0.4, 0.8 (g/kg)	Inhibit platelet activation and improves hemorheological disorders	Platelet CD36 signaling pathway (Src/ERK5/p38)	Wang et al. (2024)

### 3.2 Inhibition of platelet aggregation

In recent years, numerous pharmacological studies have demonstrated the beneficial effects of TCM in inhibiting platelet aggregation.

In the study of herbal extracts, the safflower extract compounds, such as hydroxysafflor yellow A, safflower yellow A, and luteolin, extracted from *Rheum palmatum L*., has been shown to inhibit platelet aggregation by affecting the activation of ADP receptor downstream signaling pathways, such as calcium and cAMP production, and modulating the expression of activated glycoproteins on platelet membranes (Lu et al., 2021). The extract of *Corydalis decumbens (Thunb.) Pers*. inhibits thrombosis by reducing platelet aggregation through the modulation of the PI3K-Akt pathway (Chen et al., 2024). Grape pomace extract significantly inhibits platelet aggregation (Choleva et al., 2019).

Regarding the single components of TCM, studies have shown that ginsenosides Rb2 and Rd2 in *Panax notoginseng* (Burkill) F. H. Chen ex C. H. Chow flowers play an important preventive role in thrombotic diseases by attenuating platelet activation through P2Y12-mediated cAMP/PKA and PI3K/Akt/Erk1/2 signaling (Zuo et al., 2021). Salvianolic acid B (SAB), an active ingredient in *Salvia miltiorrhiza* Bunge (SMB), inhibit platelet activation and aggregation induced by thrombin, adenosine diphosphate and collagen (Neves et al., 2024). Danshen extract (DSE), and rosmarinic acid (another active ingredient in SMB), all exert antiplatelet aggregation effects by inhibiting the enzymatic activity of endoplasmic reticulum resident protein 57 (ERp57) (Zou et al., 2018). Tetramethylpyrazine, an active ingredient in *Ligusticum sinense “Chuanxiong,”* promotes the expression of Sirtuin1 (Sirt1) and endothelial nitric oxide synthase (eNOS) by inhibiting miR-34a-5p expression and nuclear factor-κB (NF-κB) activation, thereby attenuating endothelial dysfunction and inhibiting platelet aggregation and inflammatory responses (Gao et al., 2022). Cudraxanthone B (CXB), isolated from *Morus alba* L., suppressed collagen-induced human platelet aggregation, Ca^2+^ ([Ca^2+^]i) mobilization, fibrinogen binding, fibronectin adhesion (Shin et al., 2021). While naringenin from citrus fruits suppresses platelet aggregation and arterial thrombosis by inhibiting platelet α-granule secretion, fibrinogen binding, intracellular calcium mobilization, and platelet adhesion to collagen-coated surfaces (Huang et al., 2021). Other plant extracts such as coumarins, flavonoids, alkaloids, flavonoids and anthraquinones may also exhibit antiplatelet and fibrinolytic activities (Saluk-Juszczak et al., 2010; Yoo et al., 2014; Seo et al., 2012).

In the study of Chinese herbal compound preparations (a kind of Chinese traditional medicine product that takes Chinese traditional medicine as raw material and is processed into a certain dosage form according to the prescribed prescription and preparation process under the guidance of Chinese traditional medicine theory), Xueshuantong Capsules inhibit shear-induced platelet aggregation by targeting the Piezo1 channel-mediated Ca^2+^ signaling pathway (Liu et al., 2021). The Qihuang Zhuyu formula improves coronary artery micro-thrombosis by inhibiting PI3K/Akt/αIIbβ3-mediated platelet activation and inflammatory responses (Ding et al., 2024). Danhong Injection (DHI) inhibits inflammation and platelet aggregation, reduces immune responses and peroxidation, and protects vascular endothelial and organ function, thereby preventing and treating cardiovascular disease (Bi et al., 2019). In clinical study, several researchers have found that DHI is beneficial to patients with ACS after percutaneous coronary intervention (Jing et al., 2014; Wei-hua et al., 2015; Zhuhua et al., 2016); after treating ischemic stroke patients with Qishen Quyu Formula, the platelet aggregation function indicators ADP and arachidonic acid (AA) decreased compared to before treatment (Jian-feng et al., 2023), as shown in Table 2.

**TABLE 2:** Research on TCM inhibit platelet aggregation.

Category	Source	Active chemical	Models	Doses	Effects	Pathway	Reference
Extracts	*Carthamustinctorius* L.	Safflower extracts	Platelet aggregation (induced by ADP, Fibrinogen)	10, 20, 50, 100 (μg/mL)	Promote blood circulation and remove blood stasis	ADP, cAMP, AA, and TXA2, P2Y1, P2Y12	Lu et al. (2021)
	*Corydalis decumbens* (Thunb.) Pers.	Total extract, total alkaloids	Mouse thrombus model (induced by carrageenan)	25, 50, 100 (μg/mL)	Reduce thrombus formation	PI3K/AKt	Chen et al. (2024)
	*Vitis vinifera* L.	Grape pomace	Platelet aggregation (induced by PAF, TRAP, ADP)	10 (μg/mL)	Anti-platelet effect	—	Choleva et al. (2019)
	*Salvia miltiorrhiza* Bunge.	Danshen extract (DSE)	Platelet aggregation (induced by AA, ADP)	15, 50, 150, 450, 1,350 (mg/mL)	Inhibit platelet aggregation	ERp57	Zou et al. (2018)
	*Corydalis decumbens* (Thunb.) Pers.	Tetrahydropalmatine	Platelet aggregation (induced by ADP)	25, 50, 100 (μg/mL)	Inhibit platelet aggregation	PI3K/AKt	Chen et al. (2024)
Monomer compound	*Panax notoginseng* (Burkill) F. H. Chen	Ginsenoside Rb2/Rd2	Platelet aggregation (induced by ADP, collagen, TRAP) and murine arteriole thrombosis (induced by FeCl_3_)	25, 50, 100 (μg/mL)	Inhibit platelet aggregation and arteriole thrombosis	P2Y12/CAMP/PKA, PI3K/Akt/ERK1/2	Zuo et al. (2021)
	*Citrus reticulata* Blanco.	Naringenin	Platelet aggregation (induced by ADP, thrombin and collagen)	100, 200, 400, 800 (μM)	Inhibit platelet aggregation	α particles, fibrin, Ca2+	Huang et al. (2021)
	*Salvia miltiorrhiza* Bunge.	Rosmarinic Acid	Platelet aggregation (induced by AA, ADP, collagen)	3, 10, 30, 100, 300 (μM)	Inhibit platelet aggregation	ERp57	Zou et al. (2018)
	*Carthamustinctorius* L.	Hydroxysafflor yellow A, safflower yellow A, flavonoid	Platelet aggregation (induced by ADP, Fibrinogen)	25, 100 (μM)	Promote blood circulation and remove blood stasis	ADP, cAMP, AA, and TXA2, P2Y1, P2Y12	Lu et al. (2021)
	*Salvia miltiorrhiza* Bge.	Salvianolic acid B	Platelet aggregation (induced by ADP, thrombin), intravital microscopy thrombosis model (Multiple independent injuries were induced on cremaster arterioles using microscope equipped with a pulsed nitrogen dye laser) and murine arteriole thrombosis (induced by FeCl_3_)	0.02–0.16 (mM)	Inhibit platelet activation and aggregation	Thrombin catalytic site	Neves et al. (2024)
	*Ligusticum sinense* “Chuanxiong”	Ligustrazine	Rats coronary microembolization (CME) (induced by left ventricle injection of sodium laurate)	27 (mg/kg/d)	Prevents coronary microcirculation	miR-34a-5p, sirt1, eNos, NF-κB	Gao et al. (2022)
	*Morus alba* L.	Cudraxanthone B(CXB)	Platelet aggregation (induced by collagen, thrombin)	10, 20, 30, 40 (μM)	Inhibit platelet aggregation and thrombus formation	Calcium mobilization, αIIbβ3	Shin et al. (2021)
	*Rheum palmatum* L.	Chrysophanol-8-O-glucoside	Platelet aggregation (induced by collagen, thrombin, ADP, AA)	10, 30, 100 (μM)	Inhibitory effect on rat platelet aggregation *ex vivo* and on TXA2 formation *in vitro*	Antiplatelet and anticoagulant	Seo et al. (2012)
PolyPill	Xueshuantong	—	Platelet aggregation (induced by collagen)	0.15, 0.6 (mg/mL)	Inhibit platelet aggregation	Piezo1, Ca2+	Liu et al. (2021)
	Qihuang Zhuyu formula	Calyson, Oroxin A, Protosappanin A, Kaempferol and Geniposide	Coronary microthrombosis rat model (Fan et al., 2021)	3.55, 7.1 (g/kg/day)	Inhibit platelet activation and improve coronary microthrombosis	PI3K/Akt/αIIbβ3	Ding et al. (2024)
	Dan-hong Injection	—	Blood stasis model (induced by adrenaline hydrochloride and ice water bath)	0.75, 1.5, 3 (mL/kg)	Inhibit inflammatory factors and platelet aggregation, and protect organ function	Anti-inflammation and platelet aggregation	Bi et al. (2019)

### 3.3 Inhibition of platelet contents release

Platelet release reaction refers to the process in which particles or substances stored in the lysosome of platelets are stimulated and released from the open pipeline system of platelets to the outside. These substances include P-selectin, 5-HT, ADP, Ca^2+^, B-thromboglobulin (B-TG), platelet factor 4 (PF4), etc. In addition to inhibiting platelet aggregation, TCM also play an important role in inhibiting the release of platelet contents.

In the study of herbal extracts, Fruitflow, a water-soluble tomato extract, also interferes with collagen-stimulated platelet phosphorylation of Akt/GSK3β, Syk/PLCγ2, and p38 MAPK. This is achieved by inhibiting the level of platelet TXB2, 6-keto-PGF1α and platelet factor (PF4), thereby preventing platelet activation (Chen et al., 2022).

Regarding the single components of TCM, Salvianic acid, an active ingredient in SMB, can effectively inhibits platelet activation by blocking the aggregation of reactive oxygen species (ROS) and the release of mitochondrial DNA (mtDNA) from platelets (Xue et al., 2022). Another major constituent of SMB, 15,16-dihydrotanshinone I, also significantly inhibits intracellular Ca^2+^ mobilisation, AA release, and prothrombin B2 production, demonstrating potent antiplatelet activity (Park et al., 2008). Hydroxysafflor yellow A inhibits platelet surface glycoprotein IIb/IIIa (GPIIb/IIIa) and thromboxane A2 (TXA2) expression by modulating the miR-9a-5p/SRC axis, thereby reducing platelet Ca^2+^ accumulation and subsequent platelet overactivation in rats (Huang et al., 2023). XJ-8, a flavonoid separated from Sanguis draxonis extract (extracted from *Daemonorops draco Bl.*), inhibit platelet function and thrombosis by targeting MAP3K3, which suppresses platelet dense granule release, TxA2 synthesis, and aggregation (Zhu et al., 2022). Methylsulfonylamine ginkgolide B, can significantly inhibit platelet activating factor induced Ca^2+^ release from rabbit platelets (Liu et al., 2010).

In the study of Chinese herbal compound preparations, Yiqi Huoxue Granules may inhibit thrombin-induced platelet activation by suppressing protease-activated receptor 1 (PAR-1) expression in platelets (Lei et al., 2021). Buyang Huanwu Tang can also inhibit the state of excessive platelet release, significantly reducing the release of TXB2 and β-TG (Jingqi et al., 2020). DHI also has a significant inhibitory effect on platelet aggregation. It can inhibit the synthesis process of TXA2 and effectively reduce the levels of TXB2 and plasma platelet activating factor, thereby reducing the formation of large platelet aggregation and ultimately reducing the occurrence of thrombosis (Cui et al., 2021), relevant research reports illustrated in Table 3.

**TABLE 3:** Research on TCM inhibit the release of platelet contents.

Category	Source	Active chemical	Models	Doses	Effects	Pathway	Reference
Extracts	Solanum lycopersicum	Fruitflow	Platelet aggregation (induced by collagen, ADP)	1, 3, 10, 100 (μg/mL)	Inhibit the release of platelet contents (TXB2, 6-keto-PGF1α, PF4)	Akt/GSK3β, Syk/PLCγ2 and p38 MAPK	Chen et al. (2022)
Monomer compound	*Daemonorops draco* Bl.	XJ 8	Mouse carotid artery thrombosis model induced by FeCl_3_ and platelet aggregation (induced by collagen, ADP, Fibrinogen)	20, 40, 80 (mg/kg) in rat, 5,10,20 (μM) in cell	Inhibit platelet function and thrombosis	MAP3K3, dense granule release, TxA2	Zhu et al. (2022)
	*Carthamustinctorius* L.	Hydroxysafflor yellow A	Platelet aggregation (induced by Adrenaline)	5 (μM)	Reduce the activation of platelets and inhibiting platelet aggregation	mirR-9a-5p/SRC/PLCγ2/PKC δ/MEK/ERK1/2 axis	Huang et al. (2023)
	*Salvia miltiorrhiza* Bge.	15,16-dihydrotanshinone I	Platelet aggregation (induced by collagen)	10, 100 (μM)	Inhibit rabbit platelet aggregation	Ca2+, thrombin B2, AA	Park et al. (2008)
	*Salvia miltiorrhiza* Bge.	Salvianic acid A	Platelet aggregation (induced by ADP)	50, 100,500 (μM)	Depress the collection of ROS and the release of platelet mtDNA	SIRT1/ROS/mtDNA	Xue et al. (2022)
	*Ginkgo biloba* L.	Methylsulfonylamine ginkgolide B	Platelet aggregation (induced by PAF)	1.95, 3.90, 7.80 (mg/kg)	Inhibit platelet aggregation in rabbits and reduce the release products	TXB2/6-keto-PGF1α value	Liu et al. (2010)
PolyPill	Yiqi Huoxue Granules	—	Platelet activation (induced by thrombin)	0.05, 0.1, 0.2 (mg/mL)	Inhibit PAR-1 expression and phosphorylation of ERK1/2 and p38 protein	PAR-1 protein expression	Lei et al. (2021)

## 4 Clinical application of TCM in cardiovascular diseases

TCM have a long history of use in treating cardiovascular diseases. Among them, blood-activating and blood-stasis-eliminating Chinese medicines such as Salvia divinorum, safflower, Panax ginseng and Chuanxiong rhizome are major groups of commonly used medicines with multiple advantages. Compared to single-component chemical drugs, TCM exists in the form of multiple components, offering the benefits of multi-targeting, multi-pathway actions, low adverse effects, and rich clinical practice. Modern pharmacological studies have also shown that TCM can improve thrombotic diseases by regulating platelet activation, aggregation, adhesion and granule release. Secondly, TCM treats diseases holistically, including maintaining or alleviating the disease, improving overall health, and enhancing immunity, etc. In addition to eliminating cardiac lesions, TCM can also alleviate the symptoms, slow down the progression, and effectively prevent disease recurrence. Therefore, Chinese medicine is practical and promising in the treatment of CHD. In clinical case studies, TCM extracts have also demonstrated unique advantages, such as the combination of Xueshuantong injection and Danhong injection in the clinical treatment of coronary heart disease, which can effectively improve patients’ plasma specific viscosity and whole blood viscosity, and has high safety (Li, 2023); Xuefu Zhuyu Tang can basically restore chest pain symptoms in patients with coronary heart disease, and also alleviate other accompanying symptoms (Jia and Wang, 2022); Modified Danggui Sini Tang can reduce the symptom score and Seattle Angina Questionnaire score of angina pectoris, improve hemorheological indicators, and thereby improve unstable angina pectoris in coronary heart disease (Du et al., 2022); Xuefu Zhuyu Tang can significantly reduce platelet activity units (PRU), increase coagulation formation time, coagulation reaction time, platelet inhibition rate, etc. in patients with primary stable angina pectoris (Changfu et al., 2021); Zhishi Xiebai Guizhi Decoction is effective and safe in treating CHD (Zhou, 2023). It can be seen that traditional Chinese medicine has significant therapeutic effects in improving myocardial ischemia, alleviating angina symptoms, and regulating cardiac function.

## 5 Prospects and challenges

At present, there are about 11.4 million patients with CHD in China, and its morbidity and mortality rate are rising annually, imposing an extremely high medical burden to society and the public (Lan et al., 2024). Platelets play a crucial role in the development of CHD, affecting the blood supply to the coronary arteries and promoting the disease process through various biological mechanisms. These include promoting thrombus formation, regulating inflammatory responses, inducing vasoconstriction, and altering endothelial function.

Currently, most of the commonly used antiplatelet drugs in the clinic are G protein coupled receptors inhibitors, such as the P2Y12 receptor inhibitors ticagrelor and clopidogrel, and the TXA2 cyclooxygenase inhibitor aspirin. These drugs reduce the occurrence and progression of cardiovascular diseases by inhibiting thrombus formation. However, long-term administration of these drugs can lead to adverse effects. A meta-analysis showed that the prevalence of Aspirin resistance was 52.1% and the prevalence of Clopidogrel resistance was 20.5% (Parsa-Kondelaji and Mansouritorghabeh, 2023). Additionally, the incidence of in-hospital 30 day bleeding in acute coronary syndromes ranges from 3.0% to 8.3%(Moscucci et al., 2003). Consequently, an increasing number of patients are opting for TCM treatment.

Although some progress has been made in the study of platelet activity inhibition by TCM, but the Bioavailability, the pharmacology, pharmacokinetics, adverse effects, and mechanism of action has not been thoroughly investigated due to the complexity of TCM composition. This lack of comprehensive understanding has long hindered clarity on the mechanisms through which TCM combats cardiovascular diseases. As a result, the transferability of many research results is relatively low, and the efficacy of many TCM monomers/compounds is limited to laboratory validation and has not entered clinical use. Therefore, due to the complex composition of TCM, in subsequent research, we can first study the main ingredients and targets that exert pharmacological effects from single Chinese medicinal herbs or individual components in classic formulas. Secondly, we can also explore multiple pathways simultaneously to identify more therapeutic targets. Thirdly, we can further explore the therapeutic targets of diseases by combining the characteristics of disease course and the dialectical treatment of TCM. In addition, we need to pay more attention to the combination of traditional Chinese medicine and modern medical treatment methods, and use modern pharmacological research technologies such as chips, organoids, multi omics, network pharmacology, targeted screening, molecular biology, and information science to strengthen the experimental and clinical research of TCM in various platelet related diseases. This approach will enable a more comprehensively exploration of the antiplatelet effects of Chinese medicines, as well as the material basis and mechanisms underlying their anti-cardiovascular properties.

## 6 Limitation

In addition, the sample size analyzed in this review was relatively small, we only reviewed the TCM that improves CHD through anti-platelet adhesion, anti-platelet aggregation, and anti-platelet content release. Actually, the multi-target of TCM is also reflected in its ability to resist myocardial/endothelial cell damage, inhibit inflammatory reactions, suppress oxidative stress reactions, and improve symptoms of CHD, but this is not listed in this article. We will present this part in the next article.

## 7 Conclusion

There is a close interaction between platelets and CHD, and antiplatelet therapy plays a significant role in the prevention and treatment of cardiovascular diseases. TCM exhibits notable antiplatelet activity and can regulate platelet function through specific targets and pathways, thereby intervening in the progression of CHD. Therefore, exploring new therapies against CHD from the perspective of TCM holds high application prospects.

## Glossary

CHD: Coronary heart disease
IHD: ischemic heart disease
PAD: peripheral arterial disease
TCM: Traditional Chinese Medicine
RCTs: randomised controlled trials
ACS: acute coronary syndrome
CFTR: cystic fibrosis transmembrane conductance regulator protein
SGK1: Serum/Glucocorticoid Regulated Kinase 1
ATP: Adenosine Triphosphate
ADP: Adenosine Diphosphate
PCSK9: Proprotein Convertase Subtilisin/Kexin Type 9
IL-1α: Interleukin-1α
IL-1β: Interleukin-1β
GPIIb/IIIa: Glycoprotein IIb/IIIa
5-HT: 5-hydroxytryptamine
TLR2: Toll-Like Receptor 2
CD11b: Recombinant Integrin Alpha M
MCP-1: monocyte chemotactic protein 1
ECs: endothelial cells
vWF: Willebrand factor
PLT-exo: platelet exosomes
TGFBI: transforming growth factor B-inducible
PF4: platlet factor 4
β-TG: β-thromboglobulin
P-Sel: P-selectin
PAF: P-selectin and Platelet Activating Factor
PAR-1: protease-activated receptor 1
MI: myocardial infarction
**H**_**2**_ **O**_**2**_: hydrogen peroxide
PMV: platelet microvesicles
sGC: soluble guanylate cyclase
Sirt1: Sirtuin1
eNOS: endothelial nitric oxide synthase
NF-Kβ: nuclear factor-kβ
mtDNA: mitochondrial DNA
TXA2: thromboxane A2
CAD: coronary artery disease
PODXL: Podocalyxin-Like Protein 1
VEGFA: vascular endothelial growth factor A
ERK: extracellular regulated protein kinases
AA: arachidonic acid
P2Y12: Purinergic Receptor P2Y, G Protein Coupled 12
TRAP: thrombin receptor activating peptide
