# Correction: Systematic analysis of the interaction mechanism between platelets and coronary heart disease: From molecular pathways to new strategies for plant based antiplatelet therapy

**DOI:** 10.3389/fphar.2025.1641780

**Published:** 2025-07-28

**Authors:** Li Liao, Xiaoxuan Li, Hong Xu, Jing Tang, Bo Li, Yan Tang, Fang Xie

**Affiliations:** ^1^ Pharmaceutical Department, The Second People’s Hospital of Yibin-Yibin Hospital of West China Hospital of Sichuan University, Yibin, China; ^2^ Clinical Pharmacy Department, Third Affiliated Hospital of Chengdu Medical College-Chengdu Pidu District People’s Hospital, Chengdu, China; ^3^ Department of Oncology, The Second People’s Hospital of Yibin-Yibin Hospital of West China Hospital of Sichuan University, Yibin, China

**Keywords:** coronary heart disease, platelets, thrombus, inflammation, traditional Chinese medicine

The following statement was erroneously omitted for the authors Li Liao and Xiaoxuan Li: “These authors have contributed equally to this work and share first authorship.”

The original version of this article has been updated.

